# High-Performance Liquid Chromatographic Quantification of Flavonoids in Eriocaulaceae Species and Their Antimicrobial Activity

**DOI:** 10.3390/molecules14114644

**Published:** 2009-11-16

**Authors:** Marcelo Aparecido da Silva, Claudia Andréa Lima Cardoso, Wagner Vilegas, Lourdes Campaner dos Santos

**Affiliations:** 1 UNESP-Univ Estadual Paulista, Instituto de Química, Departamento de Química Orgânica, CP 355, CEP 14800-900, Araraquara, São Paulo, Brazil; 2 UEMS, Course of Chemistry, Mato Grosso do Sul State University, CP 351, CEP 79804-970, Dourados, Mato Grosso do Sul, Brazil

**Keywords:** Eriocaulaceae, liquid chromatography, quantification, flavonoids

## Abstract

Quantification of prepared samples by analysis using high performance liquid chromatography with DAD detection was developed to analyze rutin, 6-methoxyapigenin, and 6-methoxyapigenin-7-*O*-*β-*D*-*glucopyranoside isolated from methanolic extracts of different genus: *Syngonanthus*, *Leiothix* and *Eriocaulon* (Eriocaulaceae). The linearity, accuracy, and the inter-day precision of the procedure were evaluated. The calibration curves were linear. The recoveries of the flavonoids in the samples analyzed were 96.3% to 98.5%. The percentage coefficient of variation for the quantitative analysis of the flavonoids in the analyses of the samples was under 5%. The antimicrobial activity of the five methanol extracts of these Eriocaulaceae species was assayed against the microorganisms *Staphylococcus aureus*, *Pseudomonas aeruginosa*, *Escherichia coli*, *Salmonella setubal*, *Saccharomyces cerevisiae* and *Candida albicans*. Measured MIC values ranged from 1.25 to 10.00 mg/mL. The flavonoid contents suggest that Eriocaulaceae species may be a promising source of compounds to produce natural phytomedicines.

## Introduction

Eriocaulaceae is a pantropical and predominantly herbaceous monocotyledonous family, comprising around 1,100 species in 11 genera [1]. They are frequent components of the vegetation in pools of hilly areas or swamps, especially in sandy ground areas. This family is characterized as a monophyletic taxon with the following synapomorphies: very small unisexual white flowers in dense capitulae with only one ovule per locule and having spiraperturate pollen. However, the intrafamily relationships are still unsatisfactorily resolved [2]. 

Chemical constituents have previously proven to be an important taxonomic characteristic in Eriocaulaceae. Naphthopyranones and flavonols are the major compounds of many species from the genus *Paepalanthus* [3,4]. These classes of secondary metabolites are absent in species belonging to both the *Syngonanthus* and *Leiothrix* genera. *Leiothrix* produces mainly flavones and xanthones [5], while *Syngonanthus* afforded only flavones [1,6]. From the evolutionary point of view, it is considered that *Leiothrix* and *Syngonanthus* may have been originated from *Paepalanthus*, from a more advanced position than *Eriocaulon* [1], since the substitution of flavonols by flavones [7] is a well established condition [8].

Studies aiming to describe the diverse biological activities of the Eriocaulaceae species have shown promising results. Some studies have described the antioxidant [9], cytotoxic and mutagenic activities [10,11], and some extracts of the assayed plants presented antiulcerogenic activity [12].

We report here the quantification of taxonomically relevant flavonoids rutin, 6-methoxyapigenin-7-O-β-D-glucopyranoside and 6-methoxyapigenin. These flavonoids were firstly isolated from the *Eriocaulon ligulatum* (Eriocaulaceae) [7]. The sensitivity, specificity, linearity, accuracy and inter-day precisions of the assay method were evaluated. The efficiency of each analytical procedure was assessed by calculation the recovery values. Antimicrobial activities of five methanol extracts of the the Eriocaulaceae species were assayed on six different types of microorganisms (*Staphylococcus aureus*, *Pseudomonas aeruginosa*, *Escherichia coli*, *Salmonella setubal*, *Saccharomyces cerevisiae* and *Candida albicans*).

## Results and Discussion

### HPLC Analysis

**Figure 1:**
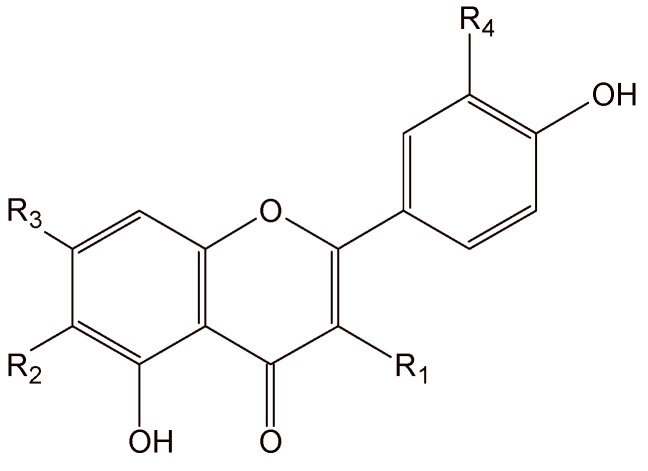
Chemical structure of the flavonoids.

In this study, three major flavonoids were detected in the methanolic extract of the different genera [*Syngonanthus*, *Leiothix* and *Eriocaulon* (Eriocaulaceae)]. A number of preliminary HPLC experiments employing samples were performed to establish optimal conditions for the analysis of rutin, 6-methoxyapigenin-7-*O*-*β-*D*-*glucopyranoside and 6-methoxyapigenin (Figure 1).

**Figure 2:**
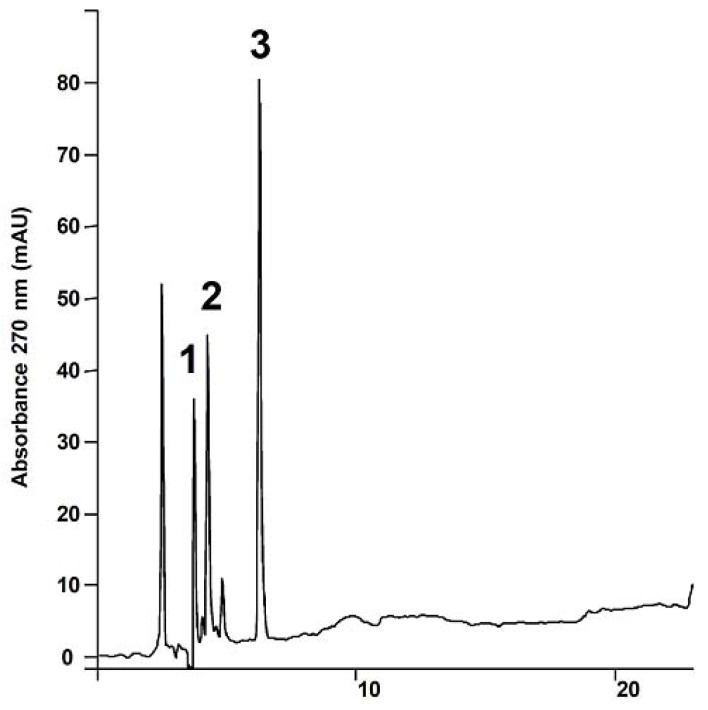
Analytical HPLC chromatogram of the compounds recorded at 270 nm: **1.** rutin, (T_r_ = 3.70 ± 0.04 min); **2.** 6-methoxyapigenin 7-*O*-*β*-D-glucopyranoside (T_r_ = 4.23 ± 0.03 min); **3.** 6-methoxyapigenin (T_r_ = 6.72 ± 0.03 min). For the chromatographic conditions, see the Experimental section.

HPLC analysis showed a baseline separation for the compounds of interest, which could be analyzed in a satisfactory time interval of less than 7 min (rutin, T_r_ = 3.70 min, 6-methoxyapigenin-7-*O*-*β-*D*-*glucopyranoside, T_r_ = 4.23 min and 6-methoxyapigenin, T_r_ = 6.72 min) (Figure 2). The intervals where the compounds were eluted were free of interference in all samples tested and were employed in sample standardization. 

The identification of rutin, 6-methoxyapigenin-7-*O*-*β-*D*-*glucopyranoside and 6-methoxyapigenin in the samples was performed by comparing their retention times with those of authentic standards and by the addition of standard solutions to the samples analyzed by HPLC and by the comparison of their UV-Vis spectrum.

There were no changes detected in the rutin, 6-methoxyapigenin-7-*O*-*β-*D*-*glucopyranoside, and 6-methoxyapigenin in the working solutions after 24 hours at 22 °C, two months at 4 °C and four months of storage at –20 °C. Thus, this validated method for the assay of flavonoids may be regarded as appropriate to indicate the stability of the solutions. The calibration curves were determined by linear regression via HPLC (Table 1). The average standard errors for the peak areas of the replicated injections (n = 5) were smaller than 1% showing good repeatability of the calibration curve.

The linearity of the method was determined by linear regression. The analysis of the samples spiked with known amounts of analyte demonstrated that the response was proportional to the concentrations of the samples with the determination coefficient being r^2^ = 0.9998 for the linear range of the analytical calibration curves for the samples.

The detection limits were 0.03 µg/mL for rutin and 0.07 µg/mL for 6-methoxyapigenin-7-*O*-*β-*D*-*glucopyranoside and 6-methoxyapigenin and the quantification limits were 0.10 µg/mL for rutin, and 0.23 µg/mL for 6-methoxyapigenin-7-*O*-*β-*D*-*glucopyranoside and 6-methoxyapigenin via HPLC. 

The recovery results showed that the procedure used is good for the extraction of the substances of interest in the samples (Table 2).

The accuracy values were less than 5% (Table 3). Regarding the precision of the assay, the inter-day coefficients of variation were less than ±5%. In this work, the precision of the method was tested for the inter-day repeatability of the samples. The inter-day variability of the assayed method was determined at low, medium and high concentrations. The results are shown in Table 3. These data indicate that the method is reproducible within three different days.

### Antimicrobial activity

The antimicrobial activity of the methanolic extracts of *L. spiralis* (**A**-capitulae, **B**-leaves), *E. ligulatum* (**C**-capitulae), *S. suberosus* (**D**-capitulae) and *S. dealbatus* (**E**-capitulae) were examined using a broth microdilution susceptibility assay against *S. aureus*, *P. aeruginosa*, *C. albicans*, *S. setubal*, *S. cerevisiae* and *E. coli*. 

Table 4 shows the contents in μg/100 mg of extract of the flavonoids employed in the HPLC method. The results presented in Table 5 reveal that the methanolic extracts inhibited the growth of all the tested microorganisms and that *C. albicans* (MIC = 1.25 mg/mL), was the most sensitive for extracts **B**, **D** and **E**, while *E. coli* was the most resistant microorganisms (MIC = 10 mg/mL) for all extracts. 

Alvarez *et al.* [13] have discussed how the important antibacterial activity of flavonoid combinations against *E.coli* forms the basis for their potential use as synergists of antibacterial agents. The authors observed that when combinations of the flavonoids kaempferol, morin, quercetin and rutin were employed, a stronger effect was found against *E. coli* than against *S. aureus*. Hidetoshi *et al.* [14] observed the antibacterial activities of flavonoids quercetin, quercetrin and morin by disk method to be enhanced by combining or mixing them. According to Harborne *et al.* [15], because of the widespread ability of flavonoids to inhibit spore germination of plant pathogens, they have been also been proposed for use against human fungal pathogens. This activity can be correlated with the one found for *C. albicans.*

In this work the flavonoids **1** and **2** are present in the methanolic extracts **A**-**E** and flavonoid **3** is present only in extracts effective against *C. albicans* at a MIC of 1.25 mg/mL suggesting that this compound is effective against this microorganism. The flavonoids combinations present antimicrobial activity against *C. albicans.* That suggests it could be an effective alternative in the treatment of infections produced by microorganisms.

## Experimental

### Plant material

Aerial parts of *Eriocaulon ligulatum* (Vell.) L.B. Smith. (voucher SANO n°2978), *Syngonanthus suberosus* Gil., (voucher SANO n°2976) *Syngonanthus dealbatus* Silv. (voucher SANO n°2975) and *Leiothrix spiralis* (Bong.) Ruhl. (voucher SANO n°4798) were collected in Diamantina, Minas Gerais State, Brazil and authenticated by Dr. Paulo Takeo Sano from the Institute of Biosciences of the University of Sao Paulo (IB-USP). A voucher specimen was deposited at the Herbarium of the IB-USP.

### Extracts preparation

Capitulae of *E. ligulatum*, *S. suberosus*, *S. dealbatus* and leaves and capitulae of *L. spiralis* were dried in an oven at 45 °C for one week and powdered separately. The resulting material (Capitulae of *E. ligulatum* (100 g), *S. suberosus* (100 g), *S. dealbatus* (100 g) and leaves and capitulae of *L. spiralis* (100 g each) were separately macerated in methanol (1 L) at room temperature for one week. The extracts were obtained after filtration and evaporation of the solvents under reduced pressure and a reduced temperature (40 °C). The yields obtained were 3.55%, 2.43%, 2.05%, 4.395 and 3.36%. 

### Reagents and standard solutions

Spectroscopy grade acetonitrile and methanol were purchased from Merck (Darmstadt, Germany). Water was purified using a Milli-Q system (Millipore). Standard substances were obtained from a collection in our laboratory. Stock mixtures of these standards were made up from individual solutions, were dissolved in methanol and then used as external standards. Standard substances were available from a collection purified by chromatography in our laboratory. The HPLC analysis for rutin (Sigma Aldrich), 6-methoxyapigenin-7-*O*-*β*-D-glucopyranoside and 6-methoxyapigenin [7], revealed a purity of 98.8%, 98.5% and 98.9%, respectively, in the standard solutions. These stock mixtures were used as external standards.

### HPLC analyses

The methanolic extracts obtained from the samples were analysed on an analytical HPLC (Varian 210) system with a ternary solvent delivery system and was equipped with an autosampler, a diode array detector (PAD) and Star WS (Workstation) software, which was used to measure the peak areas of the chromatogram. The HPLC column was an RP_18_ (25 cm × 4.6 mm × 5 μm) reversed-phase column with a small precolumn (2.5 cm × 3 mm) containing the same packing and it was used to protect the analytical column. Elution was carried out using the following program of solvent gradient: methanol:water:acetonitrile (10:80:10, v:v:v) taking 7 minutes to reach 25% methanol, 65% water and 10% acetonitrile and 5 minutes to reach 80% methanol, 10% water and 10% acetonitrile returning after that in 5 minutes to the initial conditions. The flow rate of 1.0 mL/min and an injected volume of 50 μL were used for each analysis. All chromatographic analyses were performed at 22 °C.

### Sample preparation

The prepared sample was dissolved in 2 mL of methanol, filtered through a 0.45 µm Millex filter and the resulting solution was diluted in methanol in a volumetric flask (5 mL or 10 mL) for analysis via HPLC. The validation of the procedure was carried out according to the International Conference on Harmonization Guide Lines (Validation of analytical procedures; Text on validation of analytical procedures *Q2A* and *Q2B*) [16].

### Determination of the detection and quantification limits

The detection limits were determined by injecting (n = 5) solutions of rutin, 6-methoxyapigenin-7-*O*-*β*-D-glucopyranoside and 6-methoxyapigenin of known concentration (10 µL each) and then decreasing the concentrations of the samples until a peak is detected having a signal/noise ratio of 3. The corresponding concentration was considered as being the minimal detectable concentration. The quantification limits were determined by performing the same methodology and thus, the quantification limit was considered to be the chromatographic peak having a signal/noise ratio of 10. 

### Extraction recovery

The extraction efficiency (recovery) was determined by analyzing aliquots of each sample spiked with the standards corresponding to low, medium and high concentrations. The spiked samples were submitted to the same procedure as described in “sample preparation”.

### Analytical curves

The content estimation of the rutin, 6-methoxyapigenin-7-*O-β*-D-glucopyranoside and 6-methoxy-apigenin in the samples was performed by external calibration. The compounds in the study were dissolved separately in spectroscopy grade methanol in order to obtain stock solutions, which were appropriately diluted for each of the substances. Aliquots of 10 µL dilutions for each standard were analyzed via HPLC with each determination being carried out five times. For each standard, the corresponding chromatogram was obtained and a graphical plot was constructed of the mean of areas of the chromatogram against the concentration for each substance. A linear least-square regression of the peak areas as a function of the concentrations was performed to determine the correlation coefficients. The equation parameters (slope and intercept) of each standard curve were used to obtain the concentration values for the samples. Specimens with an analytic concentration exceeding the analytical curve were reassayed upon appropriate dilution of the samples.

### Linearity

The analytical procedure was verified from the linearity of the assayed method in terms of the correlation coefficient obtained, which was evaluated by analyzing each sample (extracts **A**-**E**) spiked with known amount of the analyte at low, medium and high concentrations. The aliquots (10 µL) were analyzed via HPLC as described above. Each determination was carried out five times. For each spiked sample, the corresponding chromatograms were obtained and a plot of the average areas against their concentrations was constructed. Linear least-square regression was performed to determine the correlation coefficients. 

### Accuracy and precision

The accuracy of the assayed method was evaluated by performing replicate analyses against an analytical calibration curve and calculating the mean percentage differences between the theoretical values and the measured values. The accuracy values in the inter-day variation studies using HPLC at low, medium and high concentrations of rutin, 6-methoxyapigenin-7-*O-β*-D-glucopyranoside and 6-methoxyapigenin were evaluated in extracts **A**-**E**. The precision of a method is expressed as the percentage of the coefficient of variation (CV) of the replicate measurements. The precision of the method was tested for inter-day repeatability via HPLC. The inter-day variability of the method was determined from three different analysis (n = 5) for each sample with an addition of known amounts of analyte at low, medium and high concentrations.

### Stability study

The stability of the working standard solutions was tested at 22 °C (working temperature), 4 °C and –20 °C (storage temperatures). The stability of rutin, 6-methoxyapigenin-7-*O-β*-D-glucopyranoside and 6-methoxyapigenin in the samples was evaluated during all the storage steps (*i.e.,* at room temperature, at 4 °C and at –20 °C). Spiked samples were analyzed against the analytical calibration curves immediately after preparation (reference values) and after storage. Stability was defined as being less than 2% loss of the initial drug concentration in the stated time.

### Specificity

To evaluate the specificity of the method, two different other flavonoids (quercetin and apigenin), usually present in the genus *Syngonanthus* and *Leiothrix*, were assayed using the same HPLC procedures and the retention times of the compounds were compared with those standards in the samples.

### Antimicrobial activity

The antibacterial activity of the methanolic extract (**A** = capitulae from *L. spiralis*; **B** = leaves from *L. spiralis*; **C** = capitulae from *E. ligulatum*; **D** = capitulae from *S. suberosus*; **E** = capitulate from *S. dealbatus*) was assayed using the broth microdilution method. A collection of six microorganisms were used including a Gram-positive bacteria: *Staphylococcus aureus* (ATCC 6538p), three Gram-negative bacteria: *Escherichia coli* (ATCC 11103), *Salmonella setubal* (ATCC 19796) and *Pseudomonas aeruginosa* (ATCC 27853), two yeasts: *Saccharomyces cerevisiae* (ATCC 2601) and *Candida albicans* (ATCC 10231). Standard strains of these microorganisms were obtained from the American Type Culture Collection (ATCC), and standard antibiotics chloramphenicol and nistatine were used in order to control the sensitivity of the microbial test [17]. The minimal inhibitory concentration (MIC) was determined on 96-well culture plates using a microdilution method and a microorganism suspension at a density of 10^5^ CFU/mL in Casein Soy Broth incubated for 24 hours at 37 °C for the bacteria, and Sabouraud Broth incubated for 72 hours at 25 °C for the yeasts. Proper blanks were assayed simultaneously and the samples were tested in triplicate. Technical data have been described previously [17]. The methodology of the antibacterial activity it was standard by the National Committee for Clinical Laboratory Standards – NCCLS 2000.

## Conclusions

The HPLC analysis of the samples was developed for the simultaneous determination of rutin, 6-methoxyapigenin-7-*O*-*β-*D*-*glucopyranoside and 6-methoxyapigenin providing a method for their analysis. This method does not require tedious procedures to eliminate interfering materials. Validation experiments showed good precision and accuracy for the method with the coefficients of variation being around 1% and the relative errors being less than ± 5%. The antimicrobial activity results presented here demostrate that these methanolic extracts have phytotherapic potential against *C. albicans*.

## Figures and Tables

**Table:** 

Compounds	R_1_	R_2_	R_3_	R_4_
Rutin (**1**)	*O*-glc-(6→1)-rham	H	OH	OH
6-methoxyapigenin 7-O-*β-D-*glucopyranoside (**2**)	H	OCH_3_	O-glc	H
6-methoxyapigenin (**3**)	H	OCH_3_	OH	H

**Table 1:** Regression data of the analytical calibration curves for quantitative determination of the substances via HPLC.

Parameters	Values		
	1	2	3
LR (μg/mL)	0.10-50.00	0.23-50.00	0.23-50.00
*a*	0.0645	0.0119	0.0576
*b*	0.0133	0.0113	0.0124
*Sa*	0.0035	0.0021	0.0029
*Sb*	0.0013	0.0013	0.0010
*r*	0.9998	0.9999	0.9997
*n*	10	10	10

L.R.: linear range, *b*: slope, *a*: intercept, *Sb*: standard deviation of the slope, *Sa*: standard deviation of the intercept, *r:* correlation coefficient, *n*: number of samples. Linear regression, formula: y = a + bx, where y = ratio of peak areas, x = concentration (μg/mL), *a* = intercept and *b* = slope. Rutin (**1**); 6-methoxyapigenin-7-*O*-*β-*D*-*glucopyranoside (**2**) and 6-methoxyapigenin (**3**).

**Table 2:** Recovery of the flavonoids in samples **A**-E^*^ (n = 5).

	Rutin (%) (mean ± S.D.)				
Conc. added (μg/mL)	**A**	**B**	**C**	**D**	**E**
1	97.33 ± 1.36	97.99 ± 0.94	97.34 ± 0.99	97.45 ± 1.16	97.89 ± 0.87
20	98.79 ± 0.85	99.14 ± 1.37	98,98 ± 0.96	98.96 ± 0.78	99.44 ± 1.11
40	99.03 ± 0.97	99.05 ± 0.77	99.47 ± 1.27	99.29 ± 0.91	99.27 ± 0.80
	6-Methoxyapigenin-7-*0-β*-D-glucopyranoside (%) (mean ± S.D.)				
Conc. added (μg/mL)	**A**	**B**	**C**	**D**	**E**
1	99.45 ± 1.28	99.88 ± 0.99	99.67 ± 0.87	99.74 ± 1.25	99.68 ± 1.03
20	98.99 ± 0.73	99.35 ± 1.27	99.01 ± 1.13	98.76 ± 0.89	99.50 ± 1.19
40	98.14 ± 1.02	98.13 ± 0.97	98.00 ± 1.22	98.36 ± 1.27	98.55 ± 0.82
	6-Methoxyapigenin (%) (mean ± S.D.)				
Conc. added (μg/mL)	**A**	**B**	**C**	**D**	**E**
1	99.97 ± 0.81	99.97 ± 1.12	99.09 ± 1.19	99.89 ± 1.10	99.67 ± 0.83
20	99.43 ± 0.71	99.46 ± 0.91	99.26 ± 0.73	99.02 ± 0.86	99.29 ± 1.11
40	98.01 ± 0.67	98.39 ± 0.80	98.11 ± 1.03	98.44 ± 0.93	98.33 ± 0.76

Conc: concentration; S.D: standard deviation. **A** = Methanolic extract of the capitulae from *L. spiralis*; **B** = Methanolic extract of the leaves from *L. spiralis*; **C** = Methanolic extract of the capitulae from *E. ligulatum*; **D** = Methanolic extract of the capitulae from *S. suberosus*; **E** = Methanolic extract of the capitulae from *S. dealbatus*

**Table 3:** Inter-day accuracy and precision of the HPLC method for the determination of the flavonoids (**1**-**3**) in samples **A**-**E** (n = 5 for each sample).

	**1** (μg/mL) *			**2** (μg/mL) *			**3** (μg/mL) *		
Conc. added	Conc. found *	Ac (%)	CV (%)	Conc. found *	Ac (%)	CV (%)	Conc. found *	Ac (%)	CV (%)
1	1.03 ± 0.05	3.00	4.85	1.01 ± 0.04	1.00	3.96	1.03± 0.05	3.00	3.96
20	19.51 ± 0.53	2.45	2.72	20.02 ± 0.47	0.10	2.35	20.08 ± 0.57	0.40	2.84
40	39.32 ± 0.91	1.70	2.31	39.51± 0.85	1.23	2.15	39.43± 0,99	0.14	2.51

Conc: concentration (μg/mL); *(mean ± S.D.); CV: coefficient of variation; Ac: accuracy; S.D: standard deviations. Rutin (**1**); 6-methoxyapigenin-7-*O*-*β-*D*-*glucopyranoside (**2**) and 6-methoxyapigenin (**3**).

**Table 4:** Contents in μg/100 mg of extract **A**-**E** (mean ± S.D.) of the flavonoids (**1**-**3**) employing the HPLC method.

Extracts	1	2	3
**A**	140 ± 1.4	125 ± 1.3	--
**B**	120 ± 2.2	130 ± 1.7	150 ± 0.3
**C**	135 ± 2.4	155 ± 3.3	--
**D**	125 ± 2.2	155 ± 1.7	121 ± 0.2
**E**	130 ± 2.2	156 ± 2.1	118 ± 0.3

S.D: standard deviations; S.D: of five determinations. **A** = Methanolic extract of the capitulae from *L.spiralis*; **B** = Methanolic extract of the leaves from *L. spiralis*; **C** = Methanolic extract of the capitulae from *E. ligulatum*; **D** = Methanolic extract of the capitulae from *S. suberosus*; **E** = Methanolic extract of the capitulae from *S. dealbatus*; Rutin (**1**); 6-methoxyapigenin-7-*O*-*β-*D*-*glucopyranoside (**2)** and 6-methoxyapigenin (**3**).

**Table 5:** Antimicrobial activity of the methanolic extracts (**A**-**E**) of Eriocaulaceae genus.

	MIC^a^ (mg/mL)					
Extract^b,d^	*S. aureus*	*P. aeruginosa*	*C. albicans*	*S. setubal*	*S. cerevisiae*	*E. coli*
**A**	2.5	2.5	2.5	2.5	5	10
**B**	2.5	2.5	1.25	2.5	5	10
**C**	2.5	2.5	2.5	2.5	5	10
**D**	2.5	2.5	1.25	2.5	5	10
**E**	2.5	2.5	1.25	2.5	5	10
**Standard** ^b,c^	3 x10^-3^	3 x10^-3^	3 x10^-3^	3 x10^-3^	10x10^-3^	10 x10^-3^

^a^ Minimal inhibition concentration (MIC). ^b^Mean of 3 replicates in mg/mL. ^c^Standard antimicrobial agents: chloramphenicol against bacteria and nistatine against yeasts. ^d^**A** = Methanolic extract of the capitulae from *L.spiralis*; **B** = Methanolic extract of the leaves from *L. spiralis*; **C** = Methanolic extract of the capitulae from *E. ligulatum*; **D** = Methanolic extract of the capitulae from *S. suberosus*; **E** = Methanolic extract of the capitulae from *S. dealbatus*
